# Fires Due to Air Raids

**Published:** 1915-06-19

**Authors:** Ellis Marsland

**Affiliations:** General Hon Secretary.


*To the Editor of* The Hospital.

### Fires Due to Air Raids.

*To the Editor of* The Hospital.

Sir,—Since the issue of the British Fire Prevention Committee's "Warnings" as to the character of the fires resulting from air raids and the best means of dealing with them, many hundreds of householders have addressed themselves to this office as to whether they would be well advised to expend money on certain classes of proprietary or patented minor chemical fire appliances.

The Committee has thus deemed it to be in the public interest to make the following announcement supplemental to their previous "Warnings," more particularly as householders in their anxiety to protect their premises are frequently induced to obtain entirely unsuitable and unreliable apparatus in lieu of the more simple and economical appliances on which they can depend:

(*a*) Ordinary domestic buildings, shops, and offices should have an ample supply of buckets (kept ready filled with water), and, where funds permit, one or more small hand-pumps. Occupants should know how to use these simple appliances efficiently.

(*b*) Pitchers and other minor household vessels should be kept filled ready for use, and where there are baths, wash-tubs, etc., these should be kept filled so that a supply of water may be available independently of the water mains. Where there are garden utensils, such as syringes, garden pumps, etc., they should be readily accessible.

(*c*) On no account should money be expended on powder extinguishers (tubes), hand grenades (glass bottles or tubes), extinguishers and squirts containing carbon tetrachloride, or similar chemicals, which are liable to form noxious gases. Such appliances are unreliable or unsuitable.

(*d*) If portable liquid chemical fire extinguishers are desired, they should be of two or three gallons capacity, and a warranty should be obtained that they comply either with the specifications of the Board of Trade, H.M. Office of Works, the Metropolitan Police, or of this Committee, these specifications being mainly intended to prevent accidents from bursting.

The public should remember that fires caused by incendiary bombs, if promptly tackled with water in fair bulk, force, and continuity, need not spread, and are not quite the terrifying problem to deal with that the enemy would make them. It is mainly a matter of concentrating the available liquid fire appliances and applying water energetically so as to obtain the necessary cooling effect. The incendiary bombs so far used by the Germans do not as a rule contain petrol, but thermit, which, on combustion, takes the form of molten metal of high temperature, resultant fires having none of the characteristics of spirit fires, which latter require the use of sand.

In any event every effort should be made by the public to assist the Fire Brigades in their onerous duties, but they should not omit on any account immediately to notify any outbreak that comes to their notice by telephone or otherwise—the method of calling assistance to be posted up.

Those who desire additional useful hints on the fire question arising from possible air raids should apply for the British Fire Prevention Committee's "Warnings," which are provided gratuitously upon written application to the Registrar at 8 Waterloo Place, Pall Mall, London, S.W. (enclosing large size, stamped and addressed envelope). These "Warnings" give useful information as to the necessary precautions and how to act when the emergency arises.—Yours truly,

Ellis Marsland,
General Hon Secretary.

